# Whole Transcriptome Analysis of *Acinetobacter* *baumannii* Assessed by RNA-Sequencing Reveals Different mRNA Expression Profiles in Biofilm Compared to Planktonic Cells

**DOI:** 10.1371/journal.pone.0072968

**Published:** 2013-08-30

**Authors:** Soraya Rumbo-Feal, Manuel J. Gómez, Carmen Gayoso, Laura Álvarez-Fraga, María P. Cabral, Ana M. Aransay, Naiara Rodríguez-Ezpeleta, Ane Fullaondo, Jaione Valle, María Tomás, Germán Bou, Margarita Poza

**Affiliations:** 1 Department of Microbiology, Biomedical Research Institute, University Hospital, A Coruña, Spain; 2 Department of Molecular Evolution, Center for Astrobiology, INTA-CSIC, Madrid, Spain; 3 Genome Analysis Platform, CIC bioGUNE & CIBERehd, Derio, Spain; 4 Marine Research Division, AZTI, Tecnalia, Sukarrieta, Spain; 5 Department of Microbial biofilms, Agrobiotechnology Institute, Navarra, Spain; The Scripps Research Institute and Sorrento Therapeutics, Inc., United States of America

## Abstract

*Acinetobacter* *baumannii* has emerged as a dangerous opportunistic pathogen, with many strains able to form biofilms and thus cause persistent infections. The aim of the present study was to use high-throughput sequencing techniques to establish complete transcriptome profiles of planktonic (free-living) and sessile (biofilm) forms of *A* *. baumannii* ATCC 17978 and thereby identify differences in their gene expression patterns. Collections of mRNA from planktonic (both exponential and stationary phase cultures) and sessile (biofilm) cells were sequenced. Six mRNA libraries were prepared following the mRNA-Seq protocols from Illumina. Reads were obtained in a HiScanSQ platform and mapped against the complete genome to describe the complete mRNA transcriptomes of planktonic and sessile cells. The results showed that the gene expression pattern of *A* *. baumannii* biofilm cells was distinct from that of planktonic cells, including 1621 genes over-expressed in biofilms relative to stationary phase cells and 55 genes expressed only in biofilms. These differences suggested important changes in amino acid and fatty acid metabolism, motility, active transport, DNA-methylation, iron acquisition, transcriptional regulation, and quorum sensing, among other processes. Disruption or deletion of five of these genes caused a significant decrease in biofilm formation ability in the corresponding mutant strains. Among the genes over-expressed in biofilm cells were those in an operon involved in quorum sensing. One of them, encoding an acyl carrier protein, was shown to be involved in biofilm formation as demonstrated by the significant decrease in biofilm formation by the corresponding knockout strain. The present work serves as a basis for future studies examining the complex network systems that regulate bacterial biofilm formation and maintenance.

## Introduction

*Acinetobacter* *baumannii* are non-fermentative, oxidase negative, non-flagellated gram-negative bacilli. Although this species is a normal inhabitant of the human skin flora, intestinal tract, and respiratory system, it has been shown to cause severe disease, including bacteremia and pneumonia, especially in patients hospitalized in intensive care units and reanimation wards [1–5]. Consequently, *A* *. baumannii* was recently listed as one of the six most dangerous opportunistic pathogens [6,7]. Its high genetic plasticity allows it to rapidly adapt to stressful or otherwise unfavorable conditions by acquiring mutations, plasmids, or transposable elements. Moreover, *A* *. baumannii* species exhibit a remarkable ability to develop antibiotic resistance, which may quickly evolve into a multiresistant pattern following the acquisition of different resistance mechanisms, including β-lactamases, efflux pumps, porins, penicillin-binding proteins (PBPs), and methylase enzymes [1,2,8–17].

Bacteria often adopt sessile lifestyles in the form of matrix-enclosed habitats referred to as biofilms [18]. These are dynamic structures in which transitions between planktonic and sessile modes of growth occur in response to different environmental signals. The bacterial species inhabiting biofilms differ physiologically and behaviorally from their free-living counterparts [19]. Importantly, the structural characteristics of biofilms make them resistant to most antibiotics and host defenses [19–24]. This persistence provides a source of recurrent infections. In the case of *A* *. baumannii* , the infection of mucous surfaces and bacterial contamination of medical devices, such as intravascular catheters or endotracheal intubation, may result in biofilm formation, increasing the risk of bloodstream and respiratory infections [9].

An understanding of the ability of *A* *. baumannii* to form biofilms that adhere to and persist on a broad range of surfaces may offer the key to revealing its pathogenic mechanisms. Biofilm formation in *A* *. baumannii* has been shown to involve several regulatory processes, including those based on the sensing of bacterial cell density, the presence of different nutrients, and the concentration of free cations available to bacterial cells. Some of these extracellular signals may be sensed by two-component regulatory systems such as BfmRS. This transcriptional regulatory system activates expression of the usher-chaperone assembly apparatus responsible for the production of pili, which are needed for cell attachment and subsequent biofilm formation on polystyrene surfaces [25–27]. Recently, three new two-component sensor/regulator systems involved in biofilm formation in *P. aeruginosa* were identified [28]. In *A* *. baumannii* , a homolog of the biofilm-associated protein (Bap) of *Staphylococcus aureus* has been described [29] and the involvement of the membrane protein OmpA in the development of solid biofilms on abiotic surfaces and in virulence was demonstrated [30,31].

Several studies have reported changes in amino acid metabolism during biofilm development [32–35]. In their analyses of planktonic and sessile cells from biofilms of *A* *. baumannii* ATCC 17978, Cabral et al. [34] found differences in their proteomic profiles. In general, processes involved in bacterial adhesion and the formation and development of biofilms engage complex regulatory networks that coordinate the temporal expression of genes related to adhesion, motility, and the synthesis of matrix components. Although the ability of *A* *. baumannii* to form biofilms on abiotic surfaces contributes to the unique survival pattern of this pathogen in hospital settings, little is known about the mechanisms that promote and support biofilm formation. However, this knowledge is essential to the identification of new therapeutic targets and thus to the design of drugs effective against persistent diseases caused by multi-resistant biofilm-forming clones of *A* *. baumannii* .

Microarray technology has been used to obtain the complete transcriptional profiles of different microorganisms and offers an approach to studying biofilm formation [36,37]. For example, Whiteley et al. [38] found significant differences in gene expression between sessile and planktonic cells in *Pseudomonas aeruginosa*. Moreno-Paz et al. [19] demonstrated different profiles in cells of the iron-oxidizing bacteria *Leptospirillium* grown in biofilm vs. planktonic modes. In *A* *. baumannii* , Hood et al. described the distinct transcriptional profile of the bacterium in response to NaCl [39] while Eijkelkamp et al. [40] were able to analyze its transcriptome in cultures grown under iron-limiting conditions (which prevents biofilm formation), reporting major transcriptional changes mostly related to iron acquisition but also to motility processes.

Among the more recent techniques used to analyze the genome-wide RNA profiles of a number of organisms is deep sequencing, using the platforms 454 GS_FLX (Roche), Genome Analyzer or HiSeq (Illumina Inc.), and ABI SOLID (Life Technologies). These are open platforms not limited to the study of previously known genes, and they are sensitive as well as fast [41–48]. RNA sequencing using the Illumina system has developed as an extremely informative technique for the study of transcriptional profiles of microbes [45,49,50].

The aim of the present study was to use bacterial mRNA and the Illumina RNA-sequencing technologies to gain insight into the mechanisms behind the remarkable ability of *A* *. baumannii* to form biofilms. We therefore obtained whole transcriptomes from planktonic and biofilm cells of *A* *. baumannii* strain ATCC 17978 and then compared them for differences in their gene expression profiles.

## Materials and Methods

### Strains and culture conditions

*A* *. baumannii* ATCC 17978 was routinely grown in Mueller-Hinton (MH) broth. *E. coli* TG1, used for cloning procedures, was grown in Luria-Bertani (LB) broth. Agar was added to a final concentration of 2% when necessary. All strains were grown at 37 ^°^C with shaking (180 rpm), and stored at -80 ^°^C in LB broth containing 10% glycerol. Kanamycin (50 µg/mL) and rifampicin (50 µg/mL) were from Sigma-Aldrich (St. Louis, MO) and were added to select transformant strains. Cultures of planktonic cells originated from a single colony of *A* *. baumannii* strain ATCC 17978 isolated in MH agar and then grown in 5 mL of MH broth overnight as described above. The resulting culture was diluted 100-fold in 500 mL of MH broth in 1-L flasks and again grown as described above, measuring the optical density at 600 nm (OD600nm) every 30 min. Cells were harvested during the exponential (OD600nm = 0.4) and late stationary phases (OD600nm = 2.0) of growth, 48 h after inoculation. Planktonic and sessile cells (obtained as described below) were resuspended in RNA Later reagent (Sigma-Aldrich), frozen using liquid nitrogen, and stored at -80 ^°^C.

### Biofilm generation in Pyrex plates

*A* *. baumannii* ATCC 17978 biofilms were obtained in the Fermentation Laboratory of the Agrobiotechnology Institute (Navarra, Spain). A sample from an overnight culture of *A* *. baumannii* grown in MH broth was used to inoculate 60-mL microfermentors (Institute Pasteur, Paris, France), which were then maintained at 37 °C for 24 h. The bacterium was grown in MH broth medium under a continuous-flow culture system and continuous aeration consisting of 40 mL of compressed, sterile air/h. Submerged Pyrex slides served as the growth substratum. Biofilms that formed on the Pyrex slides were removed with a cell scraper and frozen in liquid nitrogen at -80 ^°^C.

### Isolation of mRNA

Three samples, corresponding to exponential and stationary phase cells and sessile cells from biofilms, were reduced to powder under liquid nitrogen while grinding using a mortar and pestle. Total RNA was then isolated using the mirVana miRNA isolation kit (Ambion) following the manufacturer’s protocols. Ten µg of each total RNA was further processed by removing 23S and 16S rRNAs using the MICROBExpress bacterial mRNA enrichment kit (Ambion). The rRNA-depleted samples (free of 16S and 23S rRNA) of exponentially growing, stationary phase, and biofilm cells were treated with DNAse I (Invitrogen), purified using phenol-chloroform, and concentrated by ethanol precipitation. Final concentrations and purity grades of the samples were determined using a NanoDrop ND-1000 (Thermo Scientific) and a BIOANALYZER 2100 (Agilent Technologies Inc., Germany).

### Transcription assays

A cDNA synthesis kit (Roche) was used to obtain double-stranded cDNA (ds-cDNA), following the manufacturer’s instructions. The rRNA-depleted samples together with 5’-phosphorylated degenerated hexamers and the AMV reverse transcriptase (both from Roche) were used to obtain the first cDNA strand. The second cDNA strand was then generated and treated with RNase. The ds-cDNA products were purified using the High Pure PCR purification kit (Roche). The samples were further quantified using a Nanodrop ND-1000 (Thermo Scientific) and a BIOANALYZER 2100 (Agilent Technologies Inc., Germany). The ds-cDNAs were then used in subsequent steps of the study.

### Deep-sequencing procedures

To characterize the complete transcriptomes of the studied samples, mRNA libraries from three cellular conditions (exponential and stationary phase planktonic cells and sessile cells from biofilms) were prepared following the Truseq RNA sample preparation protocols from Illumina Inc. at CIC bioGUNE’s genome analysis platform (Derio, Spain). Two biological replicates were studied for each sample.

### Read processing and comparisons of gene expression profiles

Fifty nucleotide reads from each mRNA library were obtained using HiScanSQ (Illumina Inc., CIC bioGUNE, Bilbao, Spain). Short reads were aligned against the complete genome of *A* *. baumannii* ATCC 17978 and plasmids pAB1 and pAB2 (GenBank accession codes: NC_009085.1, NC_009083.1 and NC_009084.1, respectively) using Bow tie [51], allowing a maximum of three mismatches within the first 50 bases. Reads were annotated with the R Bioconductor Genominator package and differences in expression levels estimated with the R DESeq package [52]. DESeq performs a count normalization to control the variation in the number of reads sequenced across samples. After normalization, fold changes and their significance (*p* values), indicating differential expression, were determined after a negative binomial distribution. Those mRNAs with *p* values (adjusted for a false discovery rate of 0.1%) < 0.001 were considered to be differentially expressed. Raw sequences were deposited at the NCBI Sequence Read Archive, under Bioproject accession number PRJNA191863 (experiment accessions codes SRX263965, SRX263966, SRX263968 and SRX263969 to SRX263977). Blast2GO [53] was used for the functional re-annotation of genes, the mapping of gene ontology terms, and the description of biological processes, molecular functions, cellular components, and metabolic pathways associated with the biofilm expression profiles.

### Quantitative biofilm assay

Biofilm formation was quantified using the procedure described by Tendolkar et al. [54], with slight modifications. A colony of *A* *. baumannii* was grown on MH agar medium for 18 h at 37 ^°^C and used to inoculate 25 mL of liquid MH medium, supplemented with 50 µg kanamycin /mL when necessary. The culture was maintained overnight at 37 ^°^C and 180 rpm. Cells were harvested by centrifugation (3500 *g*, 10 min), washed three times with 0.9% NaCl, and resuspended in fresh liquid medium without antibiotic. From this suspension, 100 µL (containing 10^8^ CFU) were dispensed into each well of a 96-well flat-bottom polypropylene microtiter plate containing MH medium and then incubated at 37 ^°^C for 24–48 h. Next, the cells were stained with 25 µL of a 1% w/v crystal violet solution for 15 min at room temperature, washed twice with sterile 0.9% w/v NaCl, solubilized with 200 µL of a 4: 1 v/v mixture of ethanol and acetone, and finally quantified at 570 nm. All biofilm assays were performed with at least six replicates for each strain. ANOVA tests were used to evaluate the statistical significance of the measured differences.

### Gene disruption

Plasmids were inserted into the target genes as previously described [55], with slight modifications. Briefly, kanamycin- and zeocin-resistant plasmid pCR-BluntII-TOPO (Invitrogen), unable to replicate in *A* *. baumannii* , was used as a suicide vector. An internal fragment (*~* 500 bp) of the target gene was PCR-amplified using the primers listed in Table 1 and genomic DNA from *A* *. baumannii* ATCC 17978 as template. The PCR products were cloned into the pCR-BluntII-TOPO vector and the recombinant plasmids (0.1 µg) were introduced into kanamycin- and zeocin-susceptible *A* *. baumannii* ATCC 17978 by electroporation. Mutants were selected on kanamycin-containing plates. Inactivation of the target gene by insertion of the plasmid *via* single-crossover recombination was confirmed by sequencing the PCR-amplified products using the primers listed in Table 1.

**Table 1:** Oligonucleotides and probes used in the present work.

Primer/Probe name	Sequence	Use in the present study
0114intF	actggagcgcaatcattcgt	Disruption of gene A1S_0114
0114intR	atgaagcaactccctgctgc	Disruption of gene A1S_0114
0114extF	caaggagtttgaaacgat	Confirm disruption of gene A1S_0114
0114extR	ctcgcagcaatagaccaa	Confirm disruption of gene A1S_0114
0302intF	cggaagcagtggtaaacttgc	Disruption of gene A1S_0302
0302intR	tggtgaaaacacgcgagagc	Disruption of gene A1S_0302
0302extF	acaccaactatttccgtg	Confirm disruption of gene A1S_0302
0302extR	cccaaaatcagtcaccct	Confirm disruption of gene A1S_0302
1507intF	ccacaccaactccgtttgct	Disruption of gene A1S_1507
1507intR	acttgcaaccgtgccaatga	Disruption of gene A1S_1507
1507extF	tgtgtgtgatcatttgac	Confirm disruption of gene A1S_1507
1507extR	aagagcggtttactcatc	Confirm disruption of gene A1S_1507
3168intF	atctcgagcagcttgtgcag	Disruption of gene A1S_3168
3168intR	attaagccgtggtgcaggtg	Disruption of gene A1S_3168
3168extF	actcttattgccaaaacc	Confirm disruption of gene A1S_3168
3168extR	cttgcttaatgatggagg	Confirm disruption of gene A1S_3168
2042intF	tgactggatttacacagaaga	Disruption of gene A1S_2042
2042intR	tgttccatcattaataactcc	Disruption of gene A1S_2042
2042extF	ccagagcactagccttaa	Confirm disruption of gene A1S_2042
2042extR	ttgagtgagtgcagctaa	Confirm disruption of gene A1S_2042
0114UpFNotI	cccgcggccgcgggttggtacgtgagcaactc	Construction of stable knockout strain ΔA1S_0114
0114UpRBamHI	gggggatcccccggggtaatctcctttttaacc	Construction of stable knockout strain ΔA1S_0114
0114DownFBamHI	cccggatccgggacaaccttgcacgactagaa	Construction of stable knockout strain ΔA1S_0114
0114DownRXbaI	gggtctagacccttcaagtcgacctgctacg	Construction of stable knockout strain ΔA1S_0114
pMo130 site2 F	attcatgaccgtgctgac	Confirm construction of stable knockout strain ΔA1S_0114
pMo130 site2 R	cttgtctgtaagcggatg	Confirm construction of stable knockout strain ΔA1S_0114
0114XbaIF	ccctctagaggggttattcgctcgtattgctg	Cloning of the gene A1S_0114 into the pET-RA plasmid for complementation of the stable knockout strain ΔA1S_0114
0114XbaIR	ccctctagaggggactggttgaccttcacatc	Cloning of the gene A1S_0114 into the pET-RA plasmid for complementation of the stable knockout strain ΔA1S_0114
pETRAF	ttcttcgtgaaatagtgattttt	Confirm complementation of stable knockout strain ΔA1S_0114
pETRAR	ctgtttcatatgatctgggtatc	Confirm complementation of stable knockout strain ΔA1S_0114
A1S_0109F	caaacatcgaatatccatcaatcgtc	qRT-PCR
A1S_0109R	cagccgtagatttttcaaatccg	qRT-PCR
A1S_0109 Taqman probe	cctctagcagtcaggctgtgtcatcacc	qRT-PCR
A1S_0112F	accagaagatgttggcctga	qRT-PCR
A1S_0112R	gagccgatcaaccccata	qRT-PCR
A1S_0112 Taqman Probe	gctgcctg	qRT-PCR
A1S_0113F	tggctttaacaacgctgaaa	qRT-PCR
A1S_0113R	aacccctgaccttcttcacc	qRT-PCR
A1S_0113 Taqman Probe	tgccctga	qRT-PCR
A1S_0114F	gtagagcctgagacgattgatcca	qRT-PCR
A1S_0114R	gttggctcaagttctaatttcgtca	qRT-PCR
A1S_0114 Taqman Probe	ttctaaatccccagacacagacaaagcaa	qRT-PCR
A1S_0302F	gcaggtaaagcaataatatcgaaag	qRT-PCR
A1S_0302R	ttatcaactaaggagaagctagcaagt	qRT-PCR
A1S_0302 Taqman Probe	ggaagcag	qRT-PCR
A1S_1507F	acaccaactccgtttgcttt	qRT-PCR
A1S_1507R	ctgacacttcaaatagccaggtt	qRT-PCR
A1S_1507 Taqman Probe	tcagcagc	qRT-PCR
A1S_3168F	tcgcatctcgagcagctt	qRT-PCR
A1S_3168R	cgcagctggtaattttgctt	qRT-PCR
A1S_3168 Taqman Probe	cagccacc	qRT-PCR
A1S_2042F	tggtatattgactggatttacacaga	qRT-PCR
A1S_2042R	catcattaataactccatcgagg	qRT-PCR
A1S_2042 Taqman Probe	tggctctatgagcttgttttttctatttt	qRT-PCR
gyrBF	tctctagtcaggaagtgggtacatt	qRT-PCR
gyrBR	ggttatattcttcacggccaat	qRT-PCR
gyrB Taqman Probe	tggctgtg	qRT-PCR

### Construction of knockout strains

Knockout strains were constructed using the plasmid pMo130 (Genbank accession code EU862243), containing the genes *xylE*, *sacB* and a kanamycin resistance marker, as a suicide vector [56]. Briefly, 851–932 bp upstream and downstream of the *A* *. baumannii* ATCC 17978 (Genbank accession code NC_009085.1) gene of interest were cloned into the pMo130 vector using the primers listed in Table 1. The resulting plasmid was used to transform *A* *. baumannii* by electroporation. Recombinant colonies representing the first crossover event were obtained using a combination of kanamycin selection and visual detection of XylE activity following the cathecol-based method described by Hamad et al. [56]. Bright yellow and kanamycin-resistant colonies were grown overnight in LB supplemented with 15% sucrose and then plated on the same agar medium. The second crossover event was confirmed by PCR using the primers listed in Table 1. Quantitative biofilm assays were used to determine the phenotype of the mutants.

### Complementation of stable knockout mutant

To complement the stable knockout mutant, the target gene was amplified from *A* *. baumannii* ATCC 17978 genomic DNA using the primers listed in Table 1 and then cloned into the *Xba*I restriction site of the pET-RA plasmid under the control of the β-lactamase CXT-M-14 gene promoter, as described by Aranda et al. [57]. The new construction was used to transform the mutant strain. Transformants were selected on rifampicin- and kanamycin-containing plates and confirmed by PCR using the primers listed in Table 1. The mutant strain containing the pET-RA plasmid was used as the control.

### Real-time RT-PCR

Real-time reverse transcription-PCR (RT-PCR) was carried out to determine the expression levels of a collection of genes using Taqman probes (TIB Mol Biol) listed in Table 1. In all cases, the expression levels were standardized relative to the transcription levels of the housekeeping gene *gyrB*. The primers used were those listed in Table 1. Total RNA was isolated from exponentially growing and stationary phase cultures and from the biofilms using the High Pure RNA isolation kit (Roche, Germany) and then treated with RNase-free DNase I (Invitrogen Corporation, CA). The samples were further purified using the RNeasy MinElute Cleanup kit (Qiagen, Germany). For qRT-PCR, the LightCycler 480 RNA Master hydrolysis probes kit and a LightCycler 480 RNA instrument (both from Roche, Germany) were used together with the following protocol: initial incubation of 65 ^°^C, 3 min, followed by a denaturation step at 95 ^°^C for 30 s, 45 cycles at 95 ^°^C, 15 s and 60 ^°^C, 45 s, and a final elongation step at 40 ^°^C, 30 s. All assays were performed in triplicate. The statistical significance of the determined differences was confirmed by ANOVA tests.

## Results

### Determination of the complete transcriptomes of planktonic and biofilm cells

The mRNA fractions purified from exponentially growing (Exp) and stationary-phase (Sta) cultures and from biofilms (Bio) of *A* *. baumannii* ATCC 17978 were analyzed to determine the respective gene expression level profiles and to identify differentially expressed genes. Six libraries, including two biological replicates *per* sample, were constructed (Exp 1, Exp 2, Sta 1, Sta 2, Bio 1, Bio 2) and paired-end sequenced using Illumina technology (50 bpx 2). Insert average sizes in the above mentioned libraries were 208, 240, 230, 239, 209 and 253 bp, respectively. Reads were aligned against the chromosome and plasmids of *A* *. baumannii* ATCC 17978. The number of reads that mapped against the genome is detailed in Table 2. Gene level read counts are shown in Figure S1, and MD plots and correlation between samples in Figure S2. The complete mRNA transcriptomic profiles of exponentially growing and stationary-phases cultures and from biofilm cells were obtained by Illumina procedures. Gene expression values are provided in the Supporting Information (Tables S1, S2, and S3). Table S1 shows the gene expression profile of cells obtained in exponential growth phase vs. stationary phase cultures. Table S2 and Table S3 show the gene expression profile of biofilm cells vs. exponentially growing and stationary phase cultures, respectively. Overall, the data confirmed the complete description of the whole transcriptome of each stage of growth. Approximately 97% of the genes described in the *A* *. baumannii* ATCC 17978 genome database (NC_009085.1) were transcribed using the method described herein.

**Table 2:** Total number of reads aligning with the regions of interest (coverage) of the six libraries constructed from the mRNA samples.

Exp 1	Exp 2	Sta 1	Sta 1	Bio 1	Bio 2
8189422	9497363	7707791	6317554	9084229	3111192

### Different mRNA expression patterns of cells grown in exponential phase, stationary phase and in biofilms

The expression patterns of exponentially growing vs. stationary phase cells, stationary phase cells vs. biofilm cells, and exponentially growing cells vs. biofilm cells were compared to identify differentially expressed transcripts. Up-regulated and down-regulated genes were determined based on differences for which the *p* values were below 0.001. The results are shown in Supporting Information (Tables S4, S5, S6, S7, S8, S9). Although, as expected, many genes were constitutively expressed, the comparisons indicated the association of each cellular condition with a specific expression profile, with significant differences in the expression level of a large number of genes. Thus, in biofilm vs. stationary-phase cells, 31 genes were down-regulated and 35 up-regulated; in biofilm vs. exponentially growing cells 15 genes were down-regulated and 116 up-regulated, and in stationary-phase vs. exponentially growing cells 130 genes were up-regulated and 33 down-regulated in (*p* < 0.001 in all cases).

### The gene expression profile of biofilm cells

**Figure 1:**
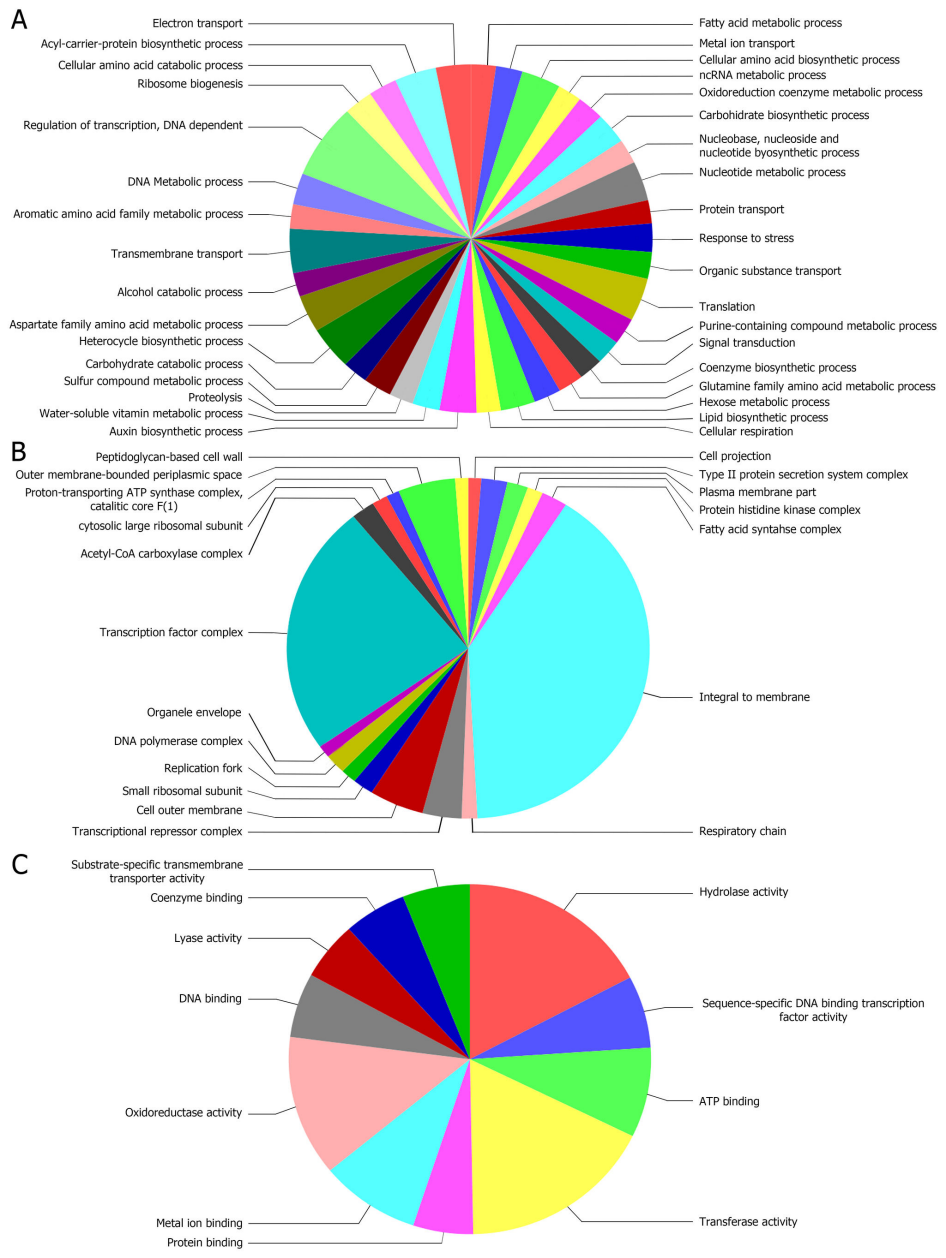
Sequence distribution of the 1621 genes identified in the present work as up-regulated in biofilm vs. stationary phase cells. Genes involved in: A) biological processes, B) cellular components, and C) molecular functions. The results were filtered by the number of sequences (cutoff = 40, 5, and 80, respectively).

A comparison of gene expression levels in biofilms vs. stationary phase cells without applying any *p* value filter indicated that among the 1621 genes over-expressed in biofilms there were 408 genes whose expression was at least four-fold higher in sessile cells or completely inhibited in planktonic cells but with an expression level value of at least 2 in biofilm. With the aim of describing gene expression profile differences in terms of gene ontology, the complete proteome of *A* *. baumannii* strain ATCC1 7978 was re-annotated using Blast2GO. Biological processes, molecular functions, and cellular components associated with the set of 1621 up-regulated genes in biofilms, as determined using Blast2GO, are shown in Figure 1. The results showed that the largest group, made up of 129 genes, was involved in transcriptional regulation. Many genes were those involved in acyl carrier protein biosynthetic processes, amino acid metabolism, fatty acid metabolism, ion transport, carbohydrate biosynthesis, translation, transmembrane transport, and the stress response, among other biological processes. The cellular location of the majority of the proteins encoded by these 1621 genes was in most cases consistent with the proteins being integral to the inner membrane or members of a transcription factor complex. Fewer proteins were located in the outer membrane periplasmic space, in the cell outer membrane, or in a transcriptional repressor complex. Moreover, there were small groups of genes that encoded proteins associated with the peptidoglycan-based cell wall, the type II protein secretion system complex, the fatty acid synthase complex, or cell projection, among other cellular components. According to the molecular function ontology, most of the genes over-expressed in biofilms were related to transferase, hydrolase, and oxidoreductase activities and, to a lesser extent, to metal ion, ATP, coenzyme, or DNA binding activities, among other molecular functions.

When the comparison of gene expression levels in biofilms *vs.* exponentially growing and stationary phase planktonic cells was filtered by *p* value (< 0.001), similar results were obtained (Figure S3). A list of genes differentially expressed (*p* < 0.001) in the biofilm vs. exponential and stationary cultures is presented in Table 3. Among them, genes coding an acyl carrier protein, an allophanate hydrolase and the RND efflux pump AdeT (A1S_0114, A1S_1278 and A1S_1755, respectively) were only expressed in biofilms and were totally inhibited in planktonic cells. Genes corresponding to hypothetical proteins (A1S_0302, A1S_0644, A1S_1293, and A1S_2893), a transmembrane arsenate pump protein (A1S_1454), the CsuD, CsuC, and CsuA/B proteins (A1S_2214, A1S_2215, and A1S_2218), the BasD protein (A1S_2382), a ferric acinetobactin binding protein (A1S_2386), a sulfate transport protein (A1S_2534), and maleylacetoacetate isomerase (A1S_3415) were also highly expressed in biofilms but totally inhibited in stationary cells. Many genes involved in amino acid metabolism and transport (such as A1S_0115, A1S_0429, A1S_1357, A1S_3134, A1S_3185, A1S_3402, A1S_3404, A1S_3405, A1S_3406, A1S_3407, or A1S_3413), or related to iron acquisition and transport (A1S_0653, A1S_0742, A1S_0980, A1S_1631, A1S_1657, A1S_2385, or A1S_2390, encoding a ferrous iron transport protein, an iron-regulated protein, a ferric enterobactin receptor, an iron-binding protein, a siderophore biosynthesis protein, a ferric acinetobactin receptor, and an acinetobactin biosynthesis protein, respectively), transcriptional regulators (A1S_1377 and A1S_1687), or encoding efflux pumps (A1S_0009 and A1S_0538) were also up-regulated in biofilm vs. planktonic cells. A gene coding for a fimbrial protein (A1S_1507) was highly up-regulated and the outer membrane protein A (A1S_2840) was down-regulated in biofilm cells vs. either exponential or stationary phase planktonic cells. An operon containing a group of genes related to phenylacetate metabolism (with identifiers A1S_1335 to A1S_1340) was up-regulated in biofilm cells vs. exponential cells but down-regulated vs. stationary cells. A homoserine lactone synthase (A1S_0109) was over-expressed in biofilms vs. either growth form of planktonic cells, as was a group of seven genes (from A1S_0112 to A1S_0118). Among the latter, A1S_0114 was an extreme case because of its very high level of expression in the biofilm (*ca.* 127) and lack of detectable expression in planktonic cells.

**Table 3:** Differentially expressed genes in biofilm-associated cells vs. both exponentially growing and stationary-phase cells.

Gene Id*	Gene description	Fold change Biofilm vs. exponential phase cells		Fold change Biofilm vs. stationary phase cells
A1S_0004	DNA gyrase		0.44	0.19
A1S_0009	RND type efflux pump		2.57	4.84
A1S_0032	signal peptide		32.18	4.82
A1S_0073	2-methylisocitrate lyase		6.60	1.61
A1S_0087	short-chain dehydrogenase		2.57	6.37
A1S_0103	3-hydroxyisobutyrate dehydrogenase		61.82	3.81
A1S_0107	enoyl-CoA hydratase		5.51	5.97
A1S_0109	homoserine lactone synthase		60.22	16.74
A1S_0112	acyl-CoA synthetase/AMP-acid ligases II		75.17	53.14
A1S_0113	acyl-CoA dehydrogenase		135.15	42.91
A1S_0114	acyl carrier protein		from zero to 127.96**	from zero to 127.96**
A1S_0115	amino acid adenylation		151.37	32.08
A1S_0116	RND superfamily exporter		56.18	79.67
A1S_0117	hypothetical protein		23.97	8.73
A1S_0118	hypothetical protein		9.31	5.26
A1S_0151	F0F1 ATP synthase subunit B		1.90	4.77
A1S_0153	F0F1 ATP synthase subunit alpha		1.13	3.75
A1S_0154	F0F1 ATP synthase subunit gamma		1.65	4.77
A1S_0155	F0F1 ATP synthase subunit beta		1.06	4.55
A1S_0156	F0F1 ATP synthase subunit epsilon		1.00	4.54
A1S_0179	NADPH-dependent FMN reductase		0.01	0.71
A1S_0279	elongation factor Tu		1.18	3.08
A1S_0283	50S ribosomal protein L11		1.99	1.94
A1S_0285	50S ribosomal protein		2.31	5.91
A1S_0292	outer membrane protein W		0.53	0.08
A1S_0302	hypothetical protein		23.30	from zero to 27.09**
A1S_0360	30S ribosomal protein S15		0.74	0.14
A1S_0429	DAACS family glutamate:aspartate symporter		3.04	1.84
A1S_0445	hypothetical protein		0.53	0.14
A1S_0449	coniferyl aldehyde dehydrogenase		0.16	0.12
A1S_0481	phosphate acetyltransferase		3.92	2.49
A1S_0482	acetate kinase		3.31	0.75
A1S_0496	phosphatidylglycerophosphatase B		10.13	1.05
A1S_0528	preprotein translocase subunit SecB		0.67	0.14
A1S_0538	RND efflux transporter		6.59	11.03
A1S_0570	hypothetical protein		0.70	0.12
A1S_0591	acyl-CoA synthetase		6.17	0.74
A1S_0628	transposase		4.32	2.80
1S_0644	hypothetical protein		18.27	from zero to 33.44**
A1S_0653	ferrous iron transport protein B		4.46	1.58
A1S_0661	phage integrase family protein		0.59	0.07
A1S_0670	protein tyrosine phosphatase		0.65	0.06
A1S_0671	protein tyrosine phosphatase		0.53	0.04
A1S_0675	dihydropteroate synthase		0.88	0.09
A1S_0736	hypothetical protein		79.49	2.76
A1S_0737	methyltetrahydropteroyltriglutamate/homocysteine S-methyltransferase		25.32	2.26
A1S_0742	iron-regulated protein		2.50	1.74
A1S_0745	hypothetical protein		30.81	4.28
A1S_0869	elongation factor Tu		1.22	2.52
A1S_0884	outer membrane protein		0.50	4.35
A1S_0971	B12-dependent methionine synthase		0.05	0.07
A1S_0980	ferric enterobactin receptor precursor		4.38	4.01
A1S_1032	hypothetical protein		4.41	2.63
A1S_1077	hypothetical protein		9.41	2.43
A1S_1104	chlorogenate esterase		0.35	0.01
A1S_1266	hypothetical protein		1.09	11.88
A1S_1278	allophanate hydrolase subunit 2		from zero to 20.35**	from zero to 20.35**
A1S_1293	hypothetical protein		15.25	from zero to 30.54**
A1S_1316	major facilitator superfamily transporter cyanate permease		14.40	4.83
A1S_1319	hypothetical protein		22.56	50.37
A1S_1335	bifunctional aldehyde dehydrogenase/enoyl-CoA hydratase		21.33	0.22
A1S_1336	phenylacetate-CoA oxygenase subunit PaaA		93.43	0.28
A1S_1337	phenylacetate-CoA oxygenase subunit PaaB		22.63	0.45
A1S_1338	hypothetical protein		34.73	0.41
A1S_1339	phenylacetate-CoA oxygenase PaaJ subunit		196.37	0.78
A1S_1340	phenylacetate-CoA oxygenase/reductase PaaK subunit		161.34	0.77
A1S_1341	enoyl-CoA hydratase/carnithine racemase		28.43	0.56
A1S_1344	thiolase		14.31	0.40
A1S_1357	alanine racemase		4.59	1.23
A1S_1370	oxidoreductase		2.67	0.80
A1S_1376	acyl-CoA dehydrogenase		11.34	3.14
A1S_1377	acrR family transcriptional regulator		4.28	0.56
A1S_1385	hypothetical protein		9.16	8.68
A1S_1454	transmembrane arsenate pump protein		54.33	from zero to 27.07**
A1S_1507	fimbrial protein		17.73	18.49
A1S_1530	SSS family major sodium/proline symporter		0.29	1.03
A1S_1541	hypothetical protein		8.27	9.19
A1S_1572	30S ribosomal protein S1		1.74	0.88
A1S_1617	30S ribosomal protein S20		4.08	1.15
A1S_1631	iron-binding protein		0.71	0.13
A1S_1637	DNA-binding protein HU-beta		1.13	5.12
A1S_1657	siderophore biosynthesis protein		13.61	2.89
A1S_1687	transcriptional regulator		1.84	0.00
A1S_1726	aspartate ammonia-lyase		0.33	0.22
A1S_1731	acetoacetyl-CoA transferase subunit beta		29.63	3.12
A1S_1732	acetoacetyl-CoA transferase subunit alpha		78.74	5.03
A1S_1736	hypothetical protein		6.21	1.47
A1S_1755	RND efflux pump AdeT		from zero to 17.27**	from zero to 17.27**
A1S_1924	cytochrome d terminal oxidase polypeptide subunit I		0.22	0.21
A1S_1925	cytochrome d terminal oxidase polypeptide subunit II		0.27	0.22
A1S_1926	hypothetical protein		0.17	0.03
A1S_1932	hypothetical protein		1.88	0.11
A1S_1965	UDP-N-acetylglucosamine acyltransferase		3.57	1.48
A1S_2072	universal stress family protein		0.52	0.21
A1S_2091	hypothetical protein		24.78	3.28
A1S_2093	hypothetical protein		1.13	0.01
A1S_2098	alcohol dehydrogenase		13.14	130.77
A1S_2102	aldehyde dehydrogenase 1		2.59	8.55
A1S_2148	acetyl-CoA synthetase/AMP-(fatty) acid ligase		12.90	0.51
A1S_2149	acyl CoA dehydrogenase oxidoreductase protein		8.68	3.11
A1S_2150	oxidoreductase		5.52	1.13
A1S_2164	phosphoenolpyruvate synthase		1.04	0.28
A1S_2183	signal peptide		0.59	0.03
A1S_2214	protein CsuD		180.04	from zero to 89.72**
A1S_2215	protein CsuC		201.23	from zero to 33.43**
A1S_2218	protein CsuA/B		164.40	from zero to 1122.03**
A1S_2261	cold shock protein		5.09	1.12
A1S_2289	signal peptide		20.61	2.63
A1S_2322	elongation factor Ts		1.50	4.89
A1S_2382	BasD (iron acquisition systems)		72.89	from zero to 24.22**
A1S_2385	ferric acinetobactin receptor		6.48	6.48
A1S_2386	ferric acinetobactin binding protein		9.10	from zero to 48.57**
A1S_2390	acinetobactin biosynthesis protein		34.11	18.97
A1S_2447	EsvD ABC transporter		7.56	14.72
A1S_2449	aromatic amino acid APC transporter		16.58	1.15
A1S_2450	pyruvate decarboxylase		8.17	0.22
A1S_2452	NAD-dependent aldehyde dehydrogenases		1.71	0.15
A1S_2458	fatty acid desaturase		0.24	0.15
A1S_2496	phosphoserine phosphatase		0.3	0.01
A1S_2534	sulfate transport protein		21.12	from zero to 24.66**
A1S_2696	hypothetical protein		1.35	0.20
A1S_2705	hypothetical protein		0.21	0.09
A1S_2718	succinyl-CoA synthetase subunit beta		1.14	7.65
A1S_2719	succinyl-CoA synthetase subunit alpha		1.07	5.76
A1S_2753	hypothetical protein		1.66	3.36
A1S_2840	outer membrane protein A		0.60	0.74
A1S_2889	signal peptide		46.50	25.85
A1S_2893	hypothetical protein		64.83	from zero to 32.31**
A1S_3043	hypothetical protein		3.91	1.5
A1S_3055	50S ribosomal protein L17		2.54	3.88
A1S_3056	DNA-directed RNA polymerase subunit alpha		1.89	3.04
A1S_3057	30S ribosomal protein S4		1.89	2.99
A1S_3058	30S ribosomal protein S11		2.00	2.58
A1S_3061	preprotein translocase subunit SecY		2.64	1.71
A1S_3062	50S ribosomal protein L15		2.79	2.07
A1S_3063	50S ribosomal protein L30		2.54	3.31
A1S_3064	30S ribosomal protein S5		2.97	3.54
A1S_3065	50S ribosomal protein L18		3.37	3.34
A1S_3066	50S ribosomal protein L6		2.55	2.29
A1S_3068	30S ribosomal protein S14		2.90	2.27
A1S_3069	50S ribosomal protein L5		2.13	1.66
A1S_3070	50S ribosomal protein L24		2.26	2.05
A1S_3073	50S ribosomal protein L29		2.01	3.00
A1S_3074	50S ribosomal protein L16		2.05	3.56
A1S_3075	30S ribosomal protein S3		1.73	3.06
A1S_3077	50S ribosomal protein L2		1.75	1.77
A1S_3079	50S ribosomal protein L4		1.87	1.59
A1S_3080	50S ribosomal protein L3		2.13	1.29
A1S_3104	ATP-dependent RNA helicase		1.64	0.23
A1S_3108	coproporphyrinogen III oxidase		0.28	0.33
A1S_3113	hypothetical protein		0.90	0.04
A1S_3134	glutamate dehydrogenase		1.26	3.21
A1S_3161	50S ribosomal protein L19		2.57	2.14
A1S_3185	glutamate synthase subunit alpha		0.40	0.48
A1S_3231	acetyl-CoA hydrolase/transferase		3.42	0.96
A1S_3297	outer membrane protein		1.17	3.80
A1S_3300	acetate permease		17.44	1.23
A1S_3301	hypothetical protein		5.77	0.52
A1S_3303	hypothetical protein		5.78	0.47
A1S_3309	acetyl-CoA synthetase		4.17	1.96
A1S_3328	pyruvate dehydrogenase subunit E1		0.50	0.92
A1S_3350	hypothetical protein		0.38	0.89
A1S_3402	arginase/agmatinase/formimionoglutamate hydrolase		3.72	7.33
A1S_3404	amino acid APC transporter		3.88	4.18
A1S_3405	histidine ammonia-lyase		3.13	3.94
A1S_3406	urocanate hydratase		3.92	0.85
A1S_3407	urocanase		4.52	2.63
A1S_3413	APC family aromatic amino acid transporter		66.30	18.38
A1S_3414	fumarylacetoacetase		60.10	20.87
A1S_3415	maleylacetoacetate isomerase		24.49	from zero to 77.30**
A1S_3416	glyoxalase/bleomycin resistance protein/dioxygenas		24.26	1.02
A1S_3418	4-hydroxyphenylpyruvate dioxygenase		78.62	12.69
A1S_3463	diaminopimelate decarboxylase		0.41	0.08
A1S_3473	hypothetical protein		0.67	0.20
A1S_3475	hypothetical protein		1.15	0.19

The data were filtered based on a *p* value < 0.001.

* Genes that significantly differed in their expression values with a *p* value below 0.001 in at least one of the two profile comparisons are listed in this table.

** In these cases the expression values are absolute and no expression was detected under planktonic conditions.

### Genes only expressed in biofilm associated cells

Fifty-five genes were exclusively expressed in sessile cells (Table 4), including 12 genes assigned to uncharacterized proteins and nine encoding transcriptional regulators (A1S_0547, A1S_1256, A1S_1430, A1S_1763, A1S_1958, A1S_2042, A1S_2151, A1S_2208 and A1S_3255). Other genes in this group belonged to the Csu operon (A1S_2216 and A1S_2217), encoded a membrane protein (A1S_0595), or were related to iron acquisition systems (A1S_0945, A1S_1719, A1S_2380, and A1S_2388). Genes coding for a DNA polymerase (A1S_2015), a DNA helicase (A1S_1585), an extracellular nuclease (A1S_1198), and an endonuclease (A1S_2408) as well as two genes involved in DNA methylation (A1S_1146 and A1S_1147) were likewise only expressed in biofilms. Other groups of genes comprised those involved in efflux systems (A1S_1117, A1S_1751, and A1S_1755), or amino acid metabolism and transport (A1S_0956 and A1S_2302), or encoded an acyl carrier protein (A1S_0114). The complete list is shown in Table 4.

**Table 4:** List of genes expressed only in biofilm cells and inhibited in planktonic cells.

Gene Id	Expression value in biofilm cells	Gene description
A1S_0079	0.47	N-acetyltransferase GNAT family (98% Ab SDF)*
A1S_0114	127.96	acyl carrier protein
A1S_0547	1.22	transcriptional regulator
A1S_0595	0.56	membrane protein (100% Ab MDR-TJ)*
A1S_0648	1.41	hypothetical protein
A1S_0741	0.19	hypothetical protein
A1S_0945	0.75	ferredoxin
A1S_0946	1.13	hypothetical protein
A1S_0956	1.13	L-aspartate dehydrogenase
A1S_0969	0.19	transketolase
A1S_1116	1.03	vanillate O-demethylase oxygenase subunit
A1S_1117	2.16	MFS superfamily vanillate transporter
A1S_1121	0.19	lipase/esterase
A1S_1125	0.38	transferase
A1S_1133	0.75	flavin-binding monooxygenase
A1S_1146	1.41	site-specific DNA-methyltransferase
A1S_1147	1.88	DNA methylase-like protein
A1S_1198	0.19	extracellular nuclease
A1S_1256	0.38	transcriptional regulator
A1S_1276	0.28	hypothetical protein
A1S_1278	20.35	allophanate hydrolase subunit 2
A1S_1349	0.47	thioesterase
A1S_1366	1.03	transporter LysE family
A1S_1430	0.28	LysR family malonate utilization transcriptional regulator
A1S_1452	0.94	arsenate reductase
A1S_1583	0.84	hypothetical protein
A1S_1585	0.56	replicative DNA helicase
A1S_1590	0.94	peptidase U35 phage prohead HK97
A1S_1622	1.13	hypothetical protein
A1S_1699	3.28	pyruvate/2-oxoglutarate dehydrogenase complex
A1S_1719	0.38	4Fe-4S ferredoxin iron-sulfur binding
A1S_1751	2.34	AdeA membrane fusion protein
A1S_1755	17.27	AdeT
A1S_1763	1.50	transcriptional regulator
A1S_1853	0.38	hypothetical protein
A1S_1887	0.28	major facilitator superfamily permease
A1S_1958	0.38	transcriptional regulator
A1S_2015	0.38	DNA-directed DNA polymerase
A1S_2028	0.19	phage putative head morphogenesis protein
A1S_2029	11.30	hypothetical protein
A1S_2033	0.47	hypothetical protein
A1S_2035	0.28	hypothetical protein
A1S_2042	2.72	transcriptional regulator (TetR family)
A1S_2151	0.75	transcriptional regulator (AraC family)
A1S_2208	0.38	transcriptional regulator
A1S_2216	11.96	CsuB
A1S_2217	3.84	CsuA
A1S_2302	0.75	ABC lysine-arginine-ornithine transporter
A1S_2380	8.81	acinetobactin biosynthesis protein
A1S_2388	1.69	putative ferric acinetobactin transport system
A1S_2408	0.09	HNH endonuclease (93% Ab MDR-TJ)*
A1S_2580	1.40	23-dihydro-2,3-dihydroxybenzoate synthetase, isochorismatase
A1S_3120	0.09	hypothetical protein
A1S_3255	0.09	transcriptional regulator AraC/XylS family protein
A1S_3260	1.59	hypothetical protein

* These genes are annotated in strain ATCC 17978. Similarities to sequences in the databases are indicated.

**Figure 2:**
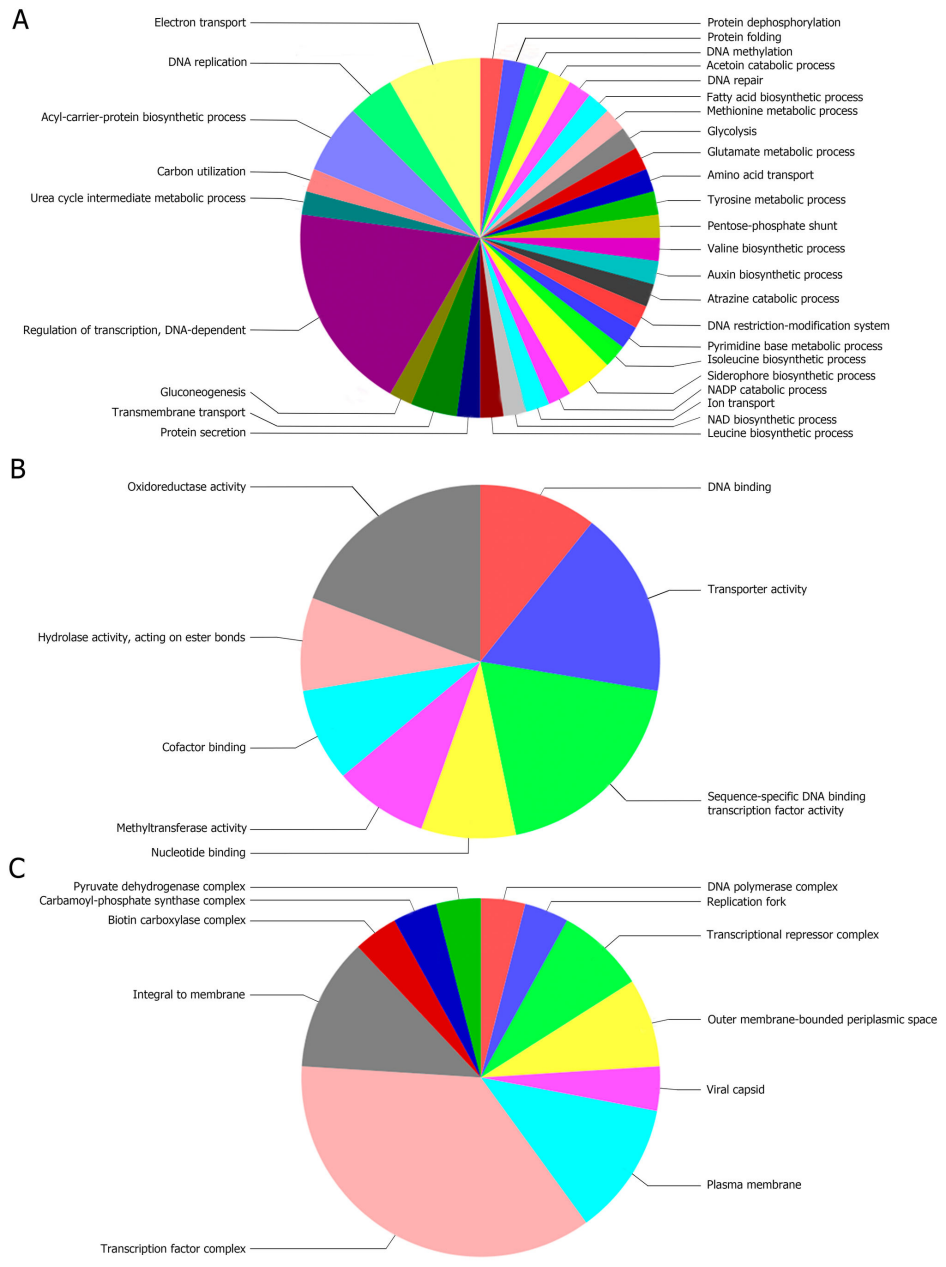
Sequence distribution of genes expressed only in biofilm-associated cells and inhibited in planktonic cells. Genes involved in A) biological processes, B) molecular functions, and C) cellular components. The results were filtered by the number of sequences (cutoff = 1, 4, and 1, respectively).

Further analysis of these 55 genes using Blast2GO revealed that most were involved in regulation of transcription and fewer to processes such as electron transport, acyl carrier biosynthesis, transmembrane transport, DNA replication, and siderophore biosynthesis (Figure 2a). The main molecular functions ascribed to the 55 genes are shown in Figure 2b, with oxidoreductase, transporters, DNA binding, and transcription factors activities predominating. The cellular location of the proteins encoded by the 55 genes is illustrated in Figure 2c, which shows that most of the proteins were located in a transcription factor complex or in the cell membrane.

### Decrease in biofilm formation ability by gene disruption and knockout mutants

**Figure 3:**
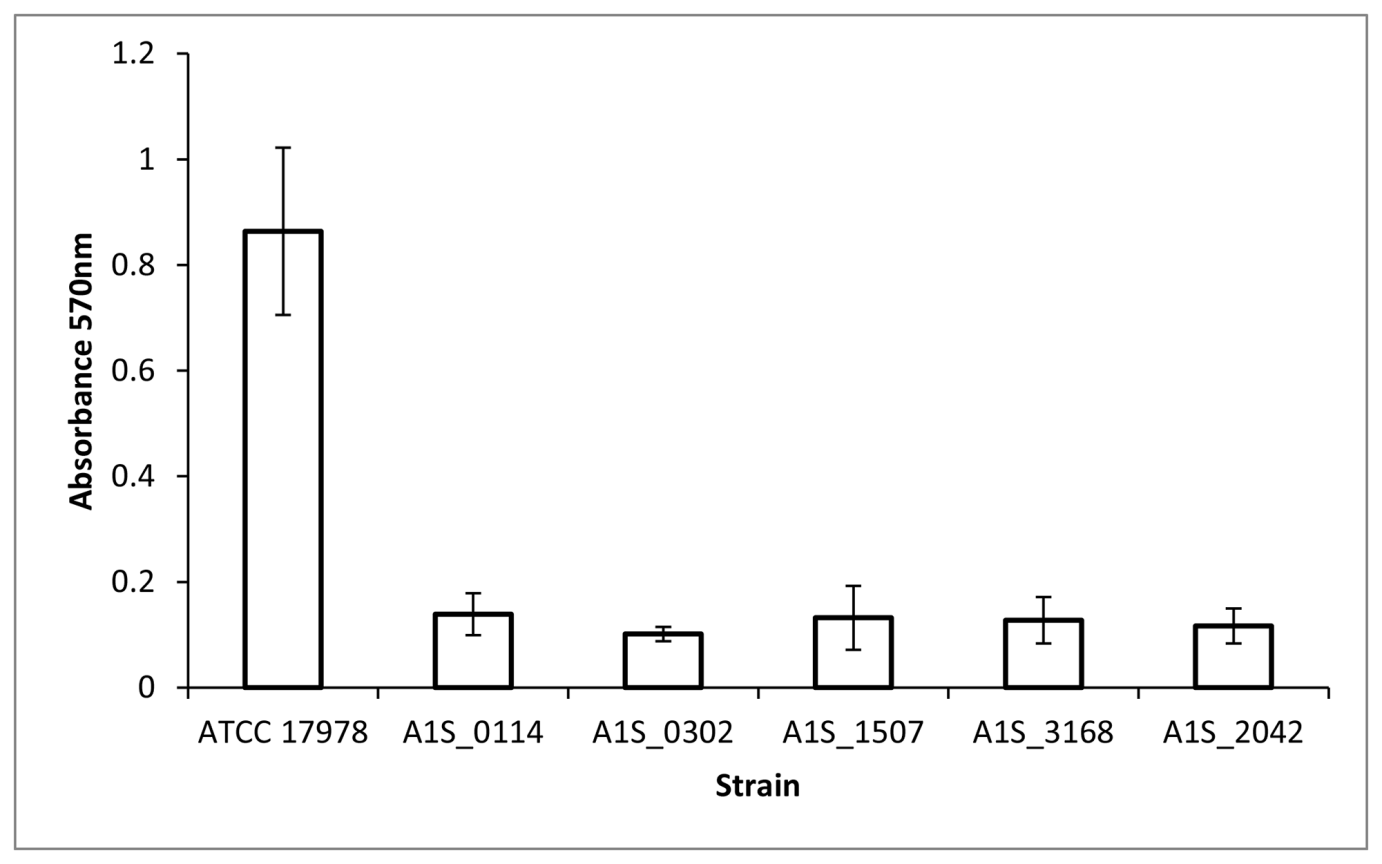
Quantification of biofilm formation by the wild-type strain (ATCC 17978) and strains with chromosomal disruptions in the genes A1S_0114, A1S_0302, A1S_1507, A1S_3168 and A1S_2042.

Five genes over-expressed in the biofilm vs. planktonic cells, as previously confirmed by qRT-PCR (Table 5), were selected for gene disruption by insertion of the plasmid pCR-Blunt-II-TOPO *via* single crossover recombination, as described in Materials and methods. These genes were A1S_0114 (encoding an acyl carrier protein expressed only in biofilms and inhibited in planktonic cells), A1S_0302 (encoding a hypothetical protein whose expression was *ca*. 27-fold higher in biofilms than in stationary-phase cells), A1S_1507 (encoding a fimbrial protein with *ca*. 18-fold higher expression in biofilms than in planktonic cells), A1S_3168 (encoding a pilus assembly protein PilW expressed in biofilms and repressed in stationary-phase cells, see Table S3), and A1S_2042 (a transcriptional regulator of the TetR family expressed in biofilms but inhibited in planktonic cells). The resulting mutant strains were used to evaluate their ability to form biofilms compared to the wild-type strain. As shown in Figure 3, biofilm formation ability was severely hindered (~8-fold reduction) in all of the mutant strains.

**Table 5:** Expression levels of genes A1S_0114, A1S_0302, A1S_1507, A1S_2042, and A1S_3168 in biofilm and planktonic cells as measured by qRT-PCR.

Gene Id	Expression level in exponential phase cells*	Expression level at the stationary phase cells	Expression level in biofilm cells
A1S_0114	1 ± 0.319	0.219 ± 0.234	6.023 ± 1.493
A1S_0302	1 ± 0.339	3.099 ± 0.847	4.069 ± 0.599
A1S_1507	1 ± 0.073	1.535 ± 0.215	7.761 ± 0.719
A1S_2042	1 ± 0.488	4.589 ± 2.152	39.35 ± 12.670
A1S_3168	1 ± 0.158	1.237 ± 0.076	2.087 ± 0.522

* The expression levels of each of the five genes were determined with respect to the exponential growth phase value, defined as 1.

**Figure 4:**
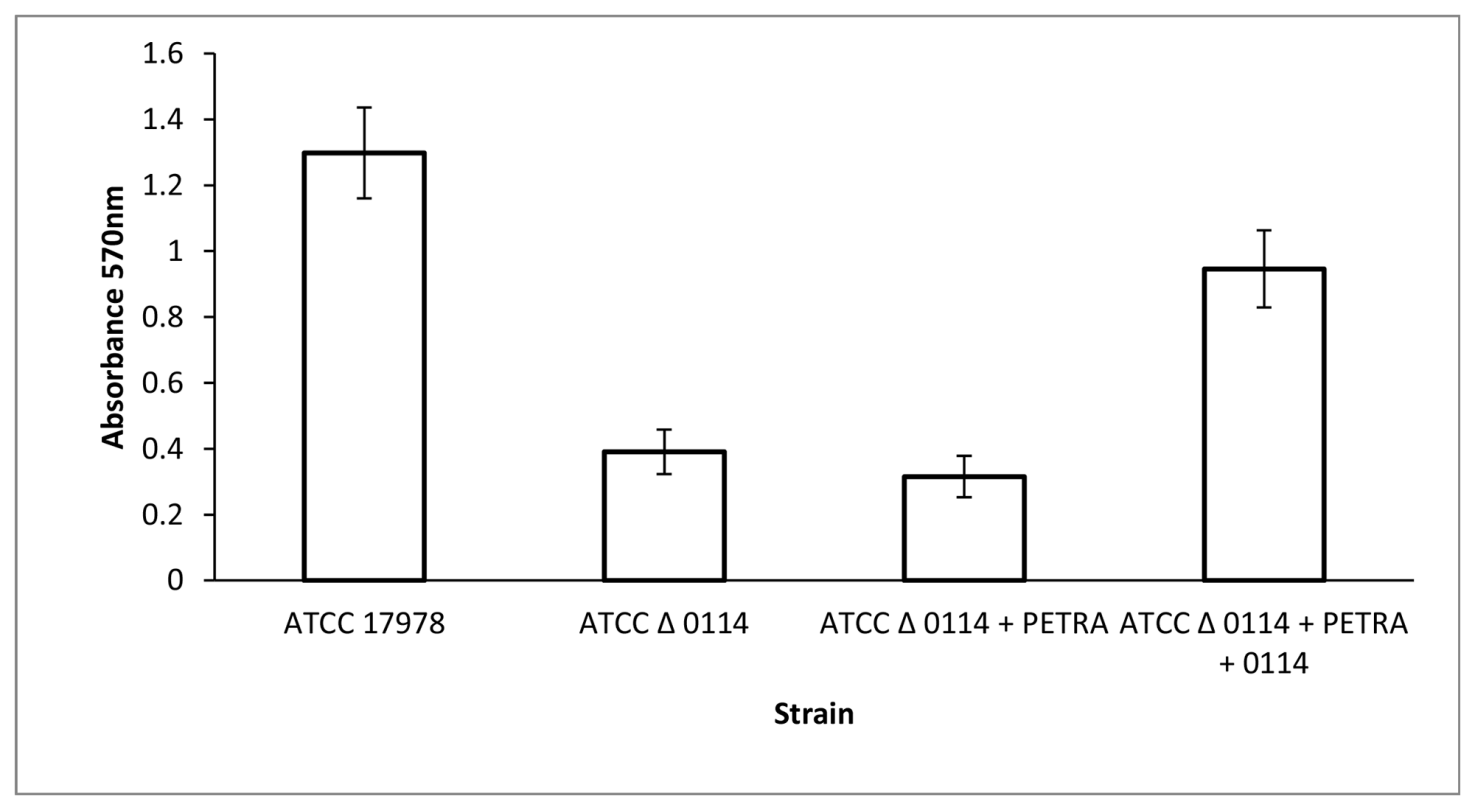
Quantification of biofilm formation by the wild-type strain (ATCC 17978), a stable knockout mutant strain lacking the gene A1S_0114 (ATCC Δ0114), the same mutant strain containing the pET-RA plasmid (ATCC Δ0114 + PETRA), and a mutant strain containing the pET-RA plasmid harboring the A1S_0114 gene (ATCC Δ0114 + PETRA + 0114).

To obtain a stable mutant free of antibiotic resistance markers or potential polar effects, the A1S_0114 gene was deleted from the genome of *A* *. baumannii* ATCC 17978 using the pMo130 vector, as described in Material and methods. As shown in Figure 4, the biofilm formation ability of the stable A1S_0114 knock-out (KO) mutant was significantly reduced (< 3-fold) compared to the wild-type strain.

**Figure 5:**
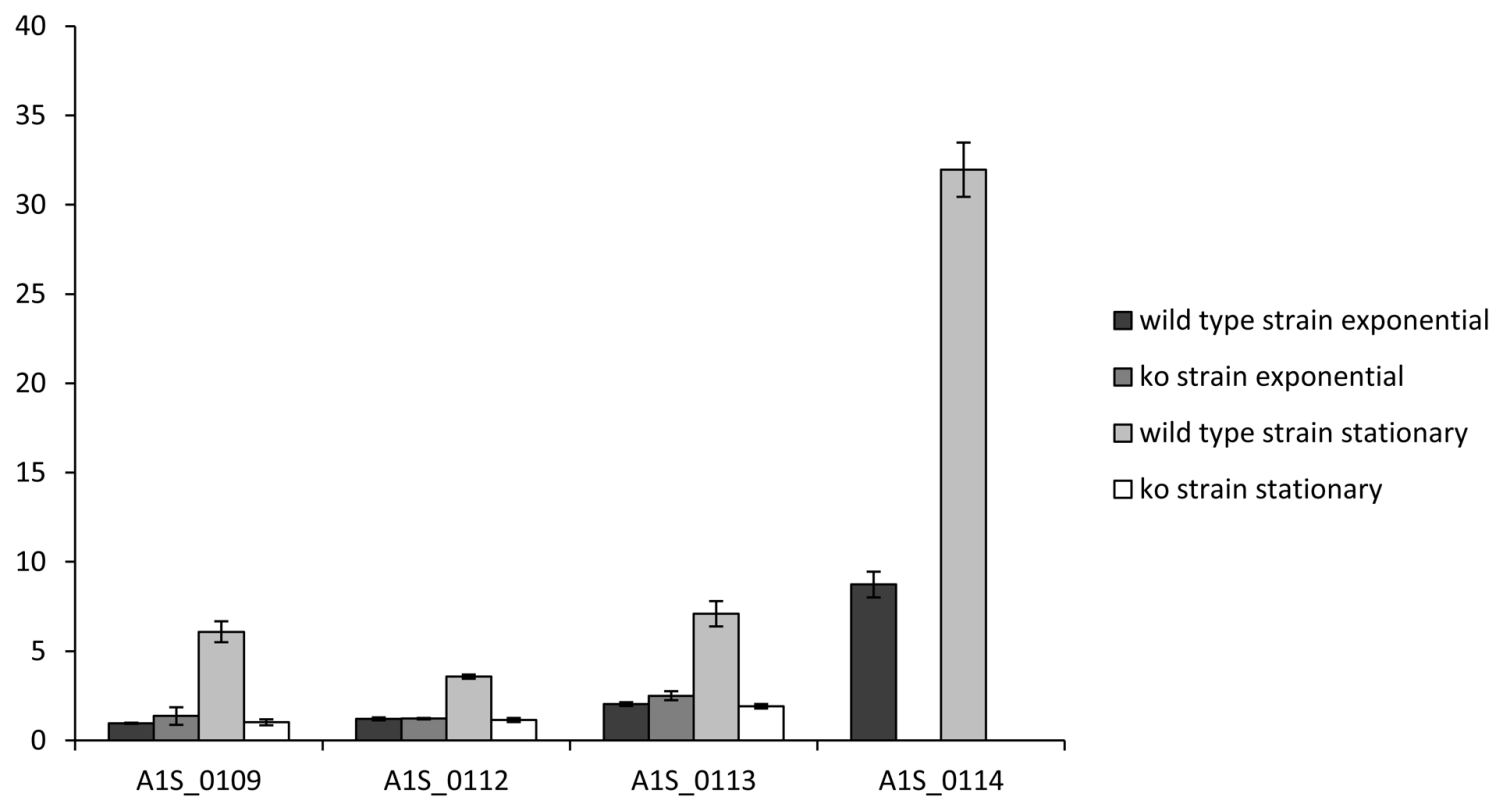
Comparison of the expression levels of genes related to homoserine lactone synthesis (A1S_0109, A1S_0112 and A1S_0113) in the wild-type strain *A* *. baumannii* 17978 and in the A1S_0114 knock-out (KO) strain as determined by real-time qRT-PCR assays.

The relationship of the gene A1S_0114 to genes related to homoserine lactone synthesis (A1S_0109, A1S_0112 and A1S_0113) was examined in qRT-PCR assays. As shown in Figure 5, genes A1S_0109, A1S_0112, A1S_0113 and A1S_0114 were over-expressed in the late stationary phase of growth compared to the exponential phase in the wild-type strain. When gene A1S_0114 was deleted from the chromosome (yielding the stable A1S_0114 KO mutant strain), the expression levels of genes related to homoserine lactone synthesis (A1S_0109, A1S_0112 and A1S_0113) were considerably reduced in the late stationary phase of growth (83, 68 and 73%, respectively) of the resulting mutant compared with the wild-type strain.

## Discussion

In the present work, we successfully used Illumina RNA-sequencing to establish the complete transcriptional profile of *A* *. baumannii* strain ATCC 17978 grown in planktonic and sessile (biofilm) modes. To obtain an overview of the temporal regulation of gene expression, planktonic cells were harvested during the exponential and late stationary phases of growth. A similar strategy was previously used in a proteomic study demonstrating the growth-dependent regulation of many proteins [58]. In another proteomic study of *A* *. baumannii* ATCC 17978 [34], planktonic and sessile cells were shown to exhibit distinct proteomic profiles, indicating that biofilms are not simply surface-attached stationary-phase cells.

Our data revealed that although many genes were constitutively expressed in both biofilm and planktonic cells, others differed in their growth-dependent expression, with clearly distinct and specific expression patterns between sessile biofilm cells and cells in either phase of planktonic growth. Among the 1621genes over-expressed in biofilms, 55 genes were only expressed in sessile cells and were totally inhibited in planktonic cells. The majority of the 55 genes encoded proteins involved in functions and mechanisms already known to be related with biofilm formation and maintenance whereas others were detected in this study for the first time. The presence of 12 genes encoding uncharacterized proteins further highlights the deficits in our knowledge of the specific genes associated with biofilm in *A* *. baumannii* . One of these genes (A1S_0302) was selected for gene disruption procedures because of its high level of expression in biofilm cells; indeed, the corresponding mutant strain was significantly deficient in biofilm formation. In addition, nine transcriptional regulators were found to be expressed only by biofilm cells, suggesting that biofilm formation and maintenance is controlled by specific molecules that are either not expressed, silenced, or not operative in planktonic cells. Gaddy and Actis [26] suggested that the regulatory process associated with biofilm formation includes the sensing of bacterial density, the presence of nutrients, and the concentration of free cations. Some of these extracellular signals are controlled by two-component regulatory systems such as BfmR/S. The *bfmS* gene encodes a sensor kinase that receives extracellular signals and phosphorylates the product of the *bfmR* gene, a response regulator. Tomaras et al. [27] studied the BfmR/S system in *A* *. baumannii* strain 19606, where this two-component regulator is required for the activation of the usher-chaperone assembly system involved in pili formation, a feature of biofilms. Based on their study of *P. aeruginosa*, Petrova et al. [59] proposed a role for BfmR in biofilm development, by limiting bacteriophage-mediated lysis and subsequent DNA release. According to our data, expression of the *bfmR* gene, identified as A1S_0748 by Liou et al. [60], was *ca*. five-fold higher in biofilm cells than in stationary cells. However, BfmR cannot be claimed as a biofilm-specific molecule of the strain 17978 since it was also expressed in the planktonic cells. No significant similarities were found in the databases for the nine transcriptional regulators described herein as biofilm specific. The mutant strain generated by the disruption of one of these genes (A1S_2042) showed an important decrease in biofilm formation ability relative to the wild-type strain. A1S_2042 appears to be a transcriptional regulator of the TetR family and could play an important role in biofilm regulation. Together with the other uncharacterized biofilm-specific transcriptional regulators, all of which were expressed at low but significant levels in biofilms, A1S_2042 merits further study to gain insight into the complex regulatory networks involved in biofilm formation and maintenance.

The CsuA/BABCDE chaperone-usher pili assembly system is involved in the adherence of *A* *. baumannii* strain 19606 biofilm to abiotic surfaces [26,27]. In the present work, Csu A/B, C, D and E were highly over-expressed in biofilms vs. planktonic cells. Of particular interest is CsuA/B, which is predicted to form part of the type I pili rod [40]. While the gene encoding CsuA/B was not expressed in stationary cells, its expression was greatly enhanced (value of 1122) in biofilms, although it was also expressed in exponentially growing cells (value of *ca* 164). Moreover, we detected CsuA (A1S_2216) and CsuB (A1S_2217) transcripts only in biofilms and not in planktonic cells. These results indicate that the complete Csu operon is highly active in the biofilms analyzed in this study. Nevertheless, it should be noted that MacQueary and Actis [61], found strong variations in the CsuA/BABCDE chaperone-usher pili assembly system and other motility factors among different strains of *A* *. baumannii* attached to abiotic surfaces. This finding may pose a challenge in the treatment of the infections caused by this bacterium, if biofilm formation on abiotic surfaces is chosen as a target for the development of new antimicrobial agents.

Two genes coding for a fimbrial protein (A1S_1507) and a pilus assembly protein PilW (A1S_3168), different from the CsuA/BABCDE chaperone-usher pili assembly system, were over-expressed in biofilm vs. planktonic cells. The disruption of these two genes in the genome of *A* *. baumannii* revealed their involvement in biofilm formation and suggested that the biofilm analyzed here could require multiple pili systems to maintain its cohesive structure. Pilus and fimbriae are important for the initial step of bacterial adhesion, which is followed by the production of exopolysaccharides, an important constituent of mature biofilms that suppresses neutrophil activity and contributes to resistance. Variation in the expression of factors involved in these pathways may account for the different capacity of bacterial strains to form biofilms and therefore to colonize or infect the host environment [62].

*A* *. baumannii* secretes a variety of molecules involved in iron acquisition including siderophores such as acinetobactin. The iron concentration in the medium acts as an important environmental signal that induces the expression of adhesion factors, thus playing a critical role in biofilm formation [63]. However, there is wide variability in the expression of iron uptake molecules, even between strains isolated during the same outbreak [61]. In our experimental model, several genes involved in iron acquisition were over-expressed in biofilm vs. planktonic cells, while some genes related to acinetobactin (A1S_2380 and A1S_2388) and ferredoxin (A1S_0945 or A1S_1719) were expressed only in biofilms and totally inhibited in planktonic cells. The exclusively expression of acinetobactin genes in biofilm cells could be explained in terms of iron starved conditions in the sessile cells compared with an iron-rich medium used for growing planktonic cells. Eijkelkamp et al. [40] found transcriptional changes in genes involved in motility when *A* *. baumannii* was grown under iron-limiting conditions. As shown by our data, the biofilm is a resistance mode where cells clearly over-express many genes related to iron acquisition systems. It is known that the ability of *A* *. baumannii* to obtain and utilize resources such as iron is an important factor for bacterial survival but it seems to also be essential for biofilm formation and maintenance, given that bacteria able to form biofilms actively search for iron [63]. This scenario was reflected in our study by the over-expression of many genes involved in iron acquisition and transport.

The detection of two genes involved in DNA methylation and expressed exclusively in biofilms suggests a role for DNA methylation in the regulation of biofilm-associated processes. In addition, several genes encoding efflux system components were activated in the biofilm cells, including the gene encoding resistance-nodulation-cell division type efflux pump (RND pump), involved in bacterial resistance to a number of antibiotics. Our results indicate that the up-regulation of efflux pumps is a mechanism of antibiotic resistance that operates in the mature biofilm [34].

Another factor previously described as involved in biofilm formation is the homolog of the staphylococcal protein Bap, studied in *A* *. baumnannii* 307-0294. The protein is a surface adhesin that mediates primary attachment to both biotic and abiotic surfaces and is involved in intercellular adhesion within the mature biofilm [29]. The *A* *. baumannii* 17978 genome (NC_009085.1) contains two loci homologous to the 5’ and 3’ ends of the *bap* locus defined in *A. baumannii* strain 307-0294 [29]. These two regions correspond to genes A1S_2724 and A1S_2696 (annotated as a hemaglutinin/hemolysin like protein and a hypothetical protein, respectively) [46] that were over-expressed in biofilms vs. exponential cells, suggesting that *A* *. baumannii* Bap-related proteins in the strain 17978 could also enhance the cell to cell interactions that support biofilm maturation.

Amino acid metabolism also clearly differed in our biofilm experimental design with respect to planktonic cells, as several genes involved in the metabolism and transport of amino acids were differentially expressed. Our results not only corroborate the hypothesis formulated by Cabral et al. [34] regarding the importance of histidine metabolism in biofilm formation but also extend it, based on our detection of genes involved in amino acid metabolism that were differentially expressed in biofilm cells and were not previously detected by proteomic analysis.

Cell surface membrane proteins may be essential to biofilm formation. Some of these proteins were differentially expressed in our biofilm cells, such as CarO (A1S_2538) and OprD-like (A1S_0201), which were up-regulated, while OmpA (A1S_2840) was down-regulated. These results conflict somewhat with those of Cabral et al. [34], who found that OmpA was up-regulated in biofilm cells. Gaddy et al. [30] also described the importance of OmpA in biofilm formation in *A* *. baumannii* strain 19606. The discrepancy in the results can be explained by strain-dependent variations or different adhesion phenomena in response to diverse biotic or abiotic surface materials, as previously described [64]. However, Marti et al. [63], analyzed the proteome of *A* *. baumannii* strain 77 and found three mass isoforms identified as OmpA. In accordance with our results, OmpA was down-regulated in the biofilm, leading the authors to suggest that this porin participates in the initiation step of biofilm formation and that the subsequent iron starvation conditions encountered during biofilm maturation trigger a decrease in its expression. This may have been the case in our experimental model. The extracellular matrix that surrounds the biolfim protects the resident bacterial cells against a number of agents but it also limits bacterial access to fresh nutrients. Accordingly, an increase in the expression of transmembrane channels may be essential for the entrance of important nutrients. In the present work, the under-expression of OmpA was complemented by an over-expression of the porins OprD-like and CarO, which may have helped to maintain the permeability of the cells in the biofilm.

Although little is known about the factors involved in biofilm regulation, cell to cell signaling mediated by N-acyl-homoserine lactones has been implicated in gram-negative bacteria [65–67]. Indeed, we identified a group of genes (identifiers A1S_0112 to A1S_0118) over-expressed in biofilms vs. planktonic cells. This group of genes has been described as an operon related to quorum sensing and may be involved in the expression of the protein encoded by A1S_0109, the only homoserine lactone synthase described thus far in *A* *. baumannii* [46,68–70]. In our experimental model, this homoserine lactone synthase (A1S_0109) was over-expressed in biofilm vs. planktonic cells. Among the genes contained in the above-mentioned operon, A1S_0114 was exclusively expressed at high levels in biofilms but totally inhibited in planktonic cells. This gene encodes a small acidic acyl carrier protein (ACP) that is very abundant in bacteria, where it serves as an important acyl donor. ACP is first synthesized in its inactive form (apo-ACP) and then activated by an acyl carrier protein synthase [71]. In its activated form, ACP is essential for the synthesis of N-acyl-homoserine lactone, which is a substrate for the homoserine lactone synthase [72]. In this work, proteins encoded by the genes A1S_0112 and A1S_0113 were identified as an acyl-CoA synthetase and an acyl-CoA dehydrogenase, respectively, both of which are necessary for ACP activation. In the gene disruption and in the stable knock out A1S_0114 (ACP) mutants there was a notable decrease in biofilm formation ability compared to the wild-type strain, demonstrating the importance of this gene in biofilm formation. Our qRT-PCR results indicated reduced expression of the genes A1S_0112, A1S_0113 as well as the N-acyl-homoserine lactone synthase gene A1S_0109 in the stable A1S_0114 KO mutant, which presumably could affect quorum sensing and biofilm formation. Moreover, since our results were consistent with alterations in fatty acid metabolism in biofilms vs. planktonic cells an alternative explanation for the decrease in biofilm formation ability of the A1S_0114 mutant is that the encoded ACP acts as an acyl donor associated with general fatty acid metabolism.

### Concluding remarks

The main goal of this study was to provide insight into the molecular mechanisms underlying the ability of *A* *. baumannii* to form biofilms. The expression profiles described herein allow the definition of many genetic elements involved in the sessile lifestyle of *A* *. baumannii* , including 55 genes exclusively expressed in biofilm. Five genes were disrupted in the chromosome and the corresponding mutant strains were significantly hindered in their biofilm formation ability, demonstrating their involvement in biofilm development. An ACP-encoding gene that belongs to an operon involved in quorum sensing mediated by a homoserine lactone was highly over-expressed in our biofilm experimental model and its inactivation significantly limited biofilm formation by cells of the corresponding mutant strain.

The results described in this work constitute a basis for the identification of new therapeutic targets and the design of new drugs able to prevent infectious diseases related to biofilm production by *A* *. baumannii* . It also serves as a starting point for future studies of the complex network systems involved in biofilm formation and maintenance, as well as the regulation of these processes.

## Supporting Information

Figure S1 **Gene level counts.** Left: boxplot (median, first and third quartiles and standard deviation) of the number of reads per gene. Right: density functions of the number of reads *per* gene.
(TIF)

Figure S2 **MD plots and correlation between samples.** Upper right: MD plots showing (countsA+countsB)/2 against (countsA-countsB), with A and B being the samples shown on the diagonal.
(TIF)

Figure S3 **Sequence distribution of genes up-regulated in biofilm-associated cells.** The data were filtered based on *p* < 0.001 and with respect to biological processes. A) Exponentially growing cells, filtered by the number of sequences (cutoff 6). B) Stationary phase cells, filtered by the number of sequences (cutoff 1).
(TIF)

Table S1 **Gene expression data from the complete transcriptome analysis of *Acinetobacter* *baumannii* ATCC 17978, showing gene expression levels in exponentially growing vs. stationary phase cells.** Id: name or code of the region of interest; baseMean: mean of the two next columns; baseMeanA: normalized number of counts for sample A; baseMeanB: normalized number of counts for sample B; Fold-change: baseMeanB/baseMeanA, log2Fold-change: log_2_ baseMeanB/baseMeanA, pval: *p* value, padj: *p* value adjusted for multiple testing, resVarA: variance of A, resVarB: variance of B.A: stationary phase cells. B: exponential phase cells. NA, non-applicable because of zero expression.
(XLSX)

Table S2 **Gene expression data from the complete transcriptome analysis of *Acinetobacter* *baumannii* ATCC 17978, showing gene expression levels in biofilm-associated cells vs. exponentially growing cells.** Id: name or code of the region of interest; baseMean: mean of the two next columns; baseMeanA: normalized number of counts for sample A; baseMeanB: normalized number of counts for sample B; Fold-change: baseMeanB/baseMeanA; log2Fold-change: log_2_ baseMeanB/baseMeanA; pval: *p* value; padj: *p* value adjusted for multiple testing; resVarA: variance of A; resVarB: variance of B.A: exponential phase cells. B: biofilm-associated cells. NA, non-applicable because of zero expression.
(XLSX)

Table S3 **Gene expression data from the complete transcriptome analysis of *Acinetobacter* *baumannii* ATCC 17978, showing gene expression levels of biofilm-associated vs. stationary phase cells.** Id: name or code of the region of interest; baseMean: mean of the two next columns; baseMeanA: normalized number of counts for sample A; baseMeanB: normalized number of counts for sample B; Fold-change: baseMeanB/baseMeanA; log2Fold-change: log_2_ baseMeanB/baseMeanA; pval: *p* value; padj: *p* value adjusted for multiple testing; resVarA: variance of A; resVarB: variance of B.A: stationary phase cells. B: biofilm-associated cells. NA: non-applicable because of zero expression.
(XLSX)

Table S4 **The expression levels of genes down-regulated in biofilm-associated vs. stationary phase cells.** The data were filtered based on *p* < 0.001. Id: name or code of the region of interest; baseMean: mean of the two next columns; baseMeanA: normalized number of counts for sample A; baseMeanB: normalized number of counts for sample B; Fold-change: baseMeanB/baseMeanA; log2Fold-change: log_2_ baseMeanB/baseMeanA; pval: *p* value; padj: *p* value adjusted for multiple testing; resVarA: variance of A; resVarB: variance of B.A: stationary phase cells. B: biofilm-associated cells.
(XLSX)

Table S5 **The expression levels of genes up-regulated in biofilm-associated vs. stationary phase cells.** The data were filtered based on *p* < 0.001. Id: name or code of the region of interest; baseMean: mean of the two next columns; baseMeanA: normalized number of counts for sample A; baseMeanB: normalized number of counts for sample B; Fold-change: baseMeanB/baseMeanA; log2Fold-change: log_2_ baseMeanB/baseMeanA; pval: *p* value; padj: *p* value adjusted for multiple testing; resVarA: variance of A; resVarB: variance of B.A: stationary phase cells. B: biofilm-associated cells.
(XLSX)

Table S6 **The expression levels of genes down-regulated in exponentially growing vs. stationary phase cells.** The data were filtered based on *p* < 0.001. Id: name or code of the region of interest; baseMean: mean of the two next columns; baseMeanA: normalized number of counts for sample A; baseMeanB: normalized number of counts for sample B; Fold-change: baseMeanB/baseMeanA; log2Fold-change: log_2_ baseMeanB/baseMeanA; pval: *p* value; padj: *p* value adjusted for multiple testing; resVarA: variance of A; resVarB: variance of B.A: stationary phase cells. B: exponential phase cells.
(XLSX)

Table S7 **The expression levels of genes up-regulated in exponentially growing vs. stationary phase cells, filtered based on *p* < 0.001.** Id: name or code of the region of interest; baseMean: mean of the two next columns; baseMeanA: normalized number of counts for sample A; baseMeanB: normalized number of counts for sample B; Fold-change: baseMeanB/baseMeanA; log2Fold-change: log_2_ baseMeanB/baseMeanA; pval: *p* value; padj: *p* value adjusted for multiple testing; resVarA: variance of A; resVarB: variance of B.A: stationary phase cells. B: exponential phase cells.
(XLSX)

Table S8 **The expression levels of genes down-regulated in biofilm-associated vs. exponentially growing cells.** The data were filtered based on *p* < 0.001. Id: name or code of the region of interest; baseMean: mean of the two next columns; baseMeanA: normalized number of counts for sample A; baseMeanB: normalized number of counts for sample B; Fold-change: baseMeanB/baseMeanA; log2Fold-change: log_2_ baseMeanB/baseMeanA; pval: *p* value; padj: *p* value adjusted for multiple testing; resVarA: variance of A; resVarB: variance of B.A: exponential phase cells. B: biofilm-associated cells.
(XLSX)

Table S9 **The expression levels of genes up-regulated in biofilm-associated vs. exponentially growing cells.** The data were filtered based on *p* < 0.001. Id: name or code of the region of interest; baseMean: mean of the two next columns; baseMeanA: normalized number of counts for sample A; baseMeanB: normalized number of counts for sample B; Fold-change: baseMeanB/baseMeanA; log2Fold-change: log_2_ baseMeanB/baseMeanA; pval: *p* value, padj: *p* value adjusted for multiple testing; resVarA: variance of A; resVarB: variance of B.A: exponential phase cells. B: biofilm-associated cells.
(XLSX)
