# Supplementary figures and images for: Whole Transcriptome Analysis of Acinetobacter baumannii Assessed by RNA-Sequencing Reveals Different mRNA Expression Profiles in Biofilm Compared to Planktonic Cells
Source: PLoS One. 2013 Aug 30;8(8):e72968. doi: 10.1371/journal.pone.0072968 (PMC3758355; doi:10.1371/journal.pone.0072968)

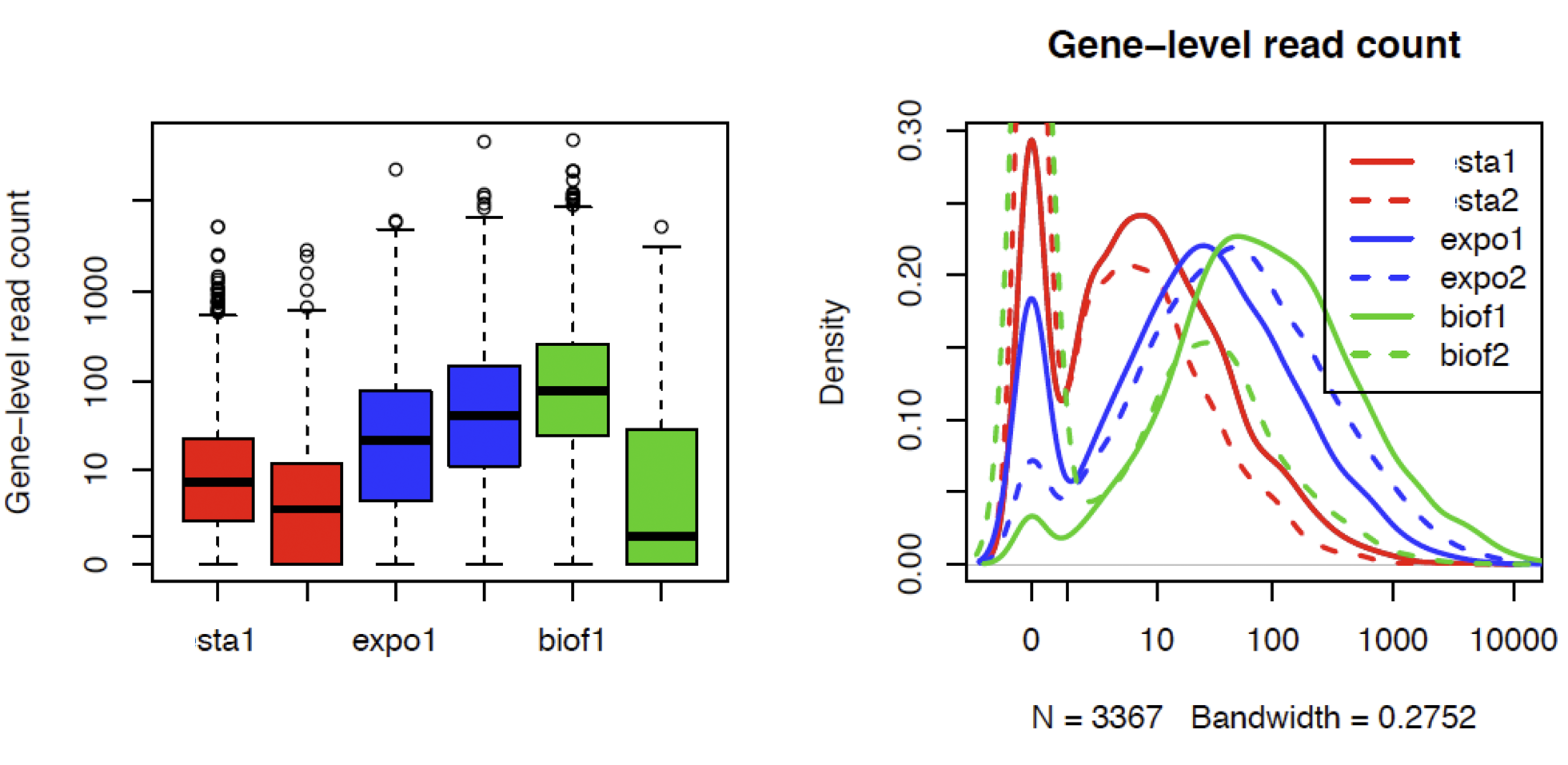

Supplement: Figure S1 — Gene level counts. Left: boxplot (median, first and third quartiles and standard deviation) of the number of reads per gene. Right: density functions of the number of reads per gene. (TIF) [file pone.0072968.s001.tif]

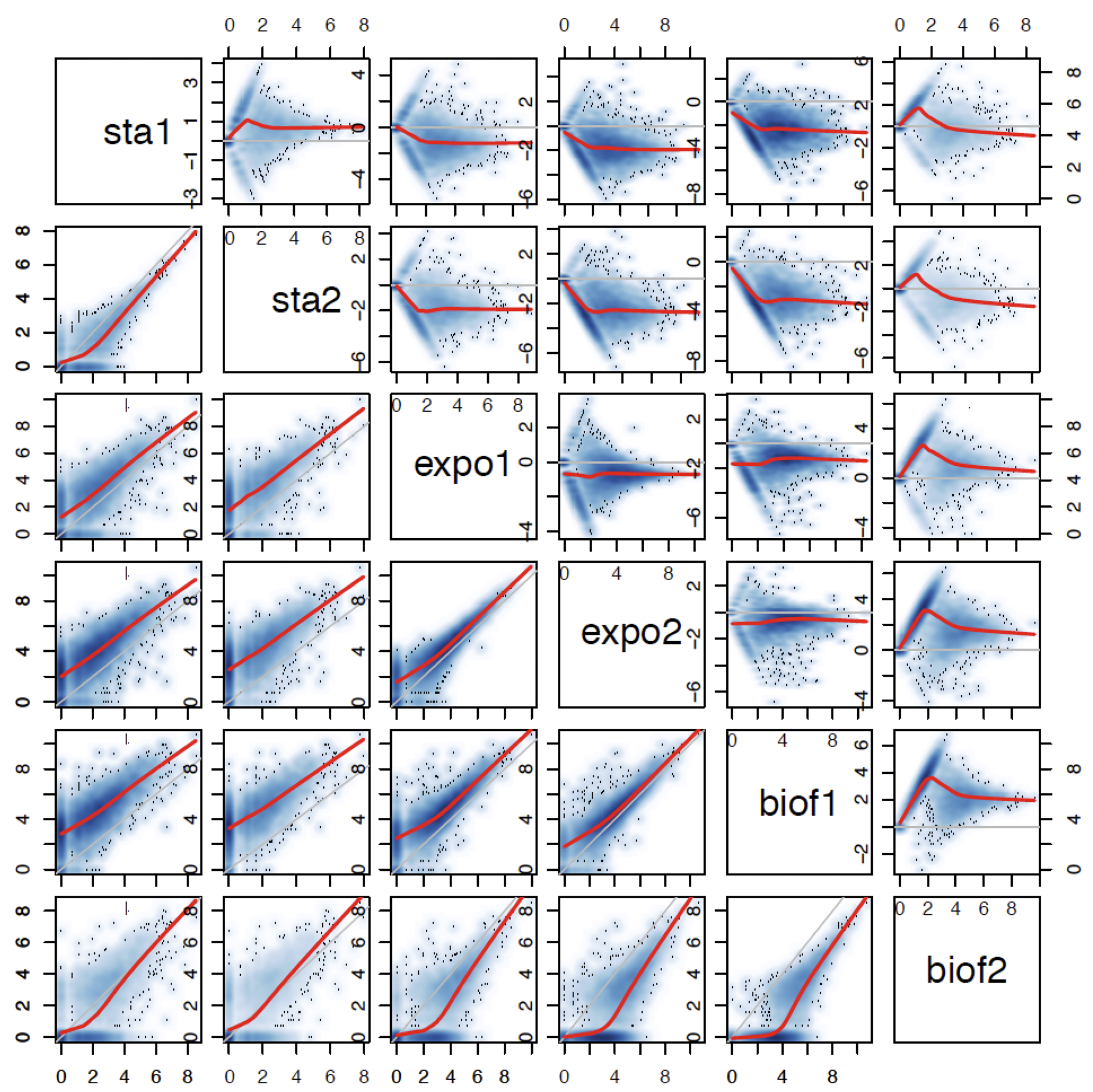

Supplement: Figure S2 — MD plots and correlation between samples. Upper right: MD plots showing (countsA+countsB)/2 against (countsA-countsB), with A and B being the samples shown on the diagonal. (TIF) [file pone.0072968.s002.tif]

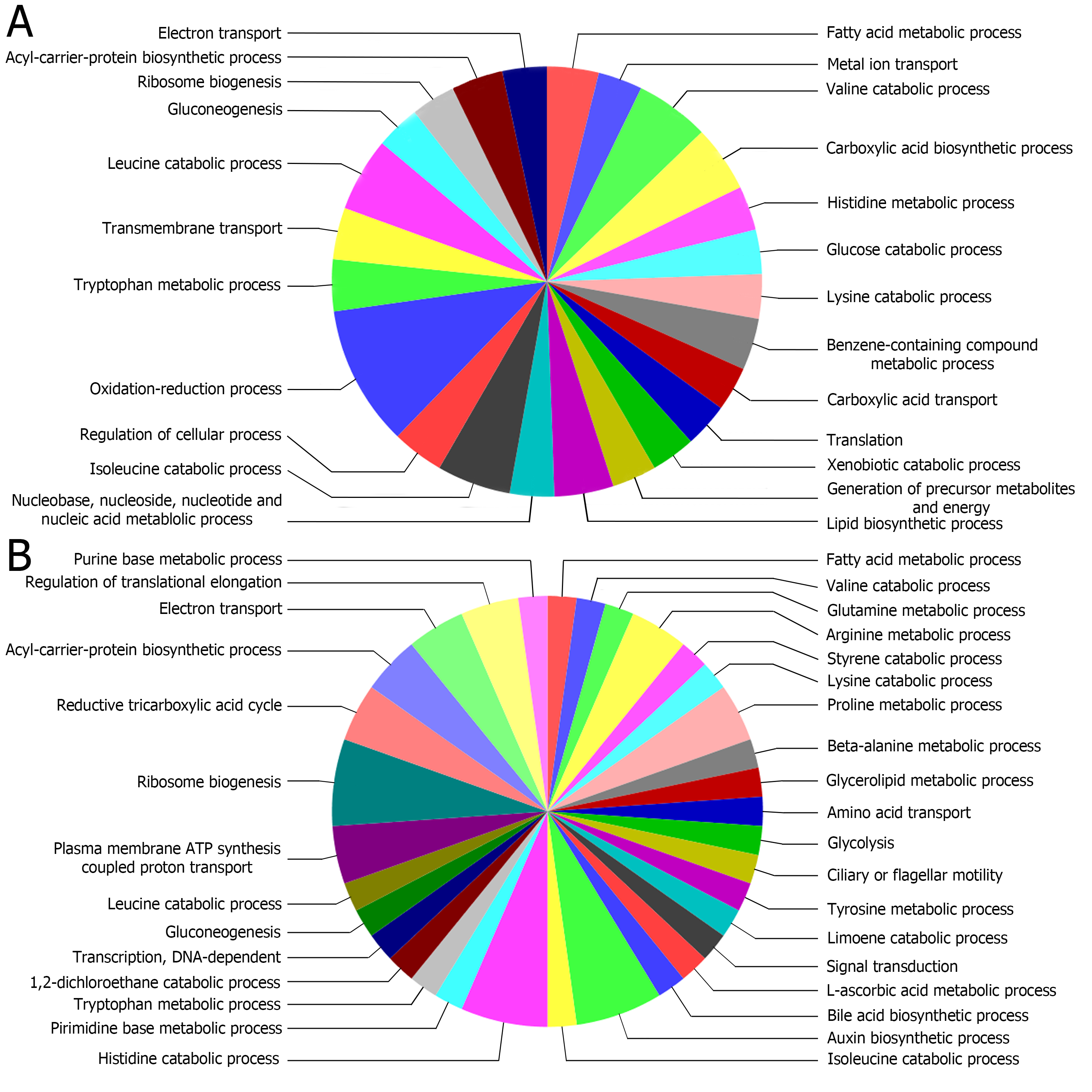

Supplement: Figure S3 — Sequence distribution of genes up-regulated in biofilm-associated cells. The data were filtered based on p < 0.001 and with respect to biological processes. A) Exponentially growing cells, filtered by the number of sequences (cutoff 6). B) Stationary phase cells, filtered by the number of sequences (cutoff 1). (TIF) [file pone.0072968.s003.tif]
